# Description of the mantle lure and novel mimicry of the endangered Cumberlandian Combshell (*Epioblasma brevidens*) in the Clinch River, eastern United States

**DOI:** 10.1002/ece3.9906

**Published:** 2023-03-24

**Authors:** Jess W. Jones, Zachary Taylor, Timothy Lane

**Affiliations:** ^1^ U.S. Fish and Wildlife Service, Department of Fish and Wildlife Conservation Virginia Tech University Blacksburg Virginia USA; ^2^ Department of Fish and Wildlife Conservation Virginia Tech University Blacksburg Virginia USA; ^3^ Aquatic Wildlife Conservation Center Virginia Department of Wildlife Resources Marion Virginia USA

**Keywords:** crayfish mimicry, Cumberlandian Combshell, *Epioblasma brevidens*, host fish capture, male mantle lure, mantle lure description

## Abstract

The Cumberlandian Combshell (*Epioblasma brevidens*) is an endangered freshwater mussel endemic to the Tennessee and Cumberland River drainages, major tributaries of the Ohio River of the eastern United States. We conducted mask and snorkel surveys in May and June of 2021 and 2022 to locate, observe, photograph, and video female *E. brevidens* to document their unique mantle lures at sites in the Clinch River in Tennessee and Virginia. The mantle lure is morphologically specialized mantle tissue that mimics prey items of the host fish. The mantle lure of *E. brevidens* appears to mimic four distinct characteristics of the reproductive anatomy of the underside (ventral) of a gravid female crayfish, to include: (1) the external apertures of the oviducts located on the base of the third pair of walking legs, (2) crayfish larvae still encased in the egg membrane, (3) pleopods or claws, and (4) postembryonic eggs. Surprisingly, we observed males of *E. brevidens* displaying mantle lures that were anatomically complex and closely resembled the female mantle lure. The male lure similarly mimics oviducts, eggs, and pleopods but is diminutive (2–3 mm smaller in length or diameter) to those same structures in females. We describe for the first time the mantle lure morphology and mimicry of *E. brevidens*, showing its close resemblance to the reproductive anatomy of a gravid female crayfish, and a novel form of mimicry in males. To our knowledge, mantle lure displays in males have not been previously documented in freshwater mussels.

## Graphical abstract

We describe for the first time the mantle lure morphology and mimicry of *Epioblasma brevidens*, showing its close resemblance to the reproductive anatomy of a gravid female crayfish, and a novel form of mimicry in males. The mantle lure is morphologically specialized mantle tissue that mimics prey items of the host fish. The mantle lure of *E. brevidens* appears to mimic four distinct characteristics of the reproductive anatomy of the underside (ventral) of a gravid female crayfish, to include: (1) the external apertures of the oviducts located on the base of the third pair of walking legs, (2) crayfish larvae still encased in the egg membrane, (3) pleopods or claws, and (4) postembryonic eggs. Surprisingly, we observed males of *E. brevidens* displaying mantle lures that were anatomically complex and closely resembled the female mantle lure.

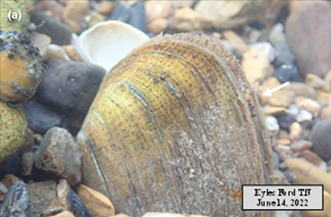

## 1 INTRODUCTION

The Cumberlandian Combshell (*Epioblasma brevidens*) is an endangered freshwater mussel species endemic to the Tennessee and Cumberland River drainages, major tributaries of the Ohio River of the eastern United States. Listed by the U.S. Fish and Wildlife Service (USFWS) as an endangered species in 1997 under the U.S. Endangered Species Act (1973), *E. brevidens* only has one remaining stronghold, a large population of >50,000 individuals in the Clinch River in Tennessee (TN) and Virginia (VA) (Ahlstedt et al., 2016; Jones et al., 2014, 2018; Jones & Neves, 2011; Lane et al., 2021; USFWS, 2004). Smaller native populations occur in the Powell River in eastern TN, a tributary to the Clinch River, Bear Creek in Alabama (AL), and the Big South Fork Cumberland River, TN, and Kentucky (KY). Several additional populations are being restored to suitable habitats in the reaches of the upper Clinch River in Russell County, VA, and the lower Powell River and Nolichucky rivers in TN (Hyde & Jones, 2021). However, despite these conservation actions to restore populations, >95% of the historical abundance and distribution of the species has been lost over the last 100 years or more (USFWS, 2004). Knowledge of the reproductive biology and life history of *E. brevidens* would be helpful to biologists working to recover the species and for the public to understand the biological complexity and importance of conserving these animals (FMCS, 2016).

**FIGURE 1:**
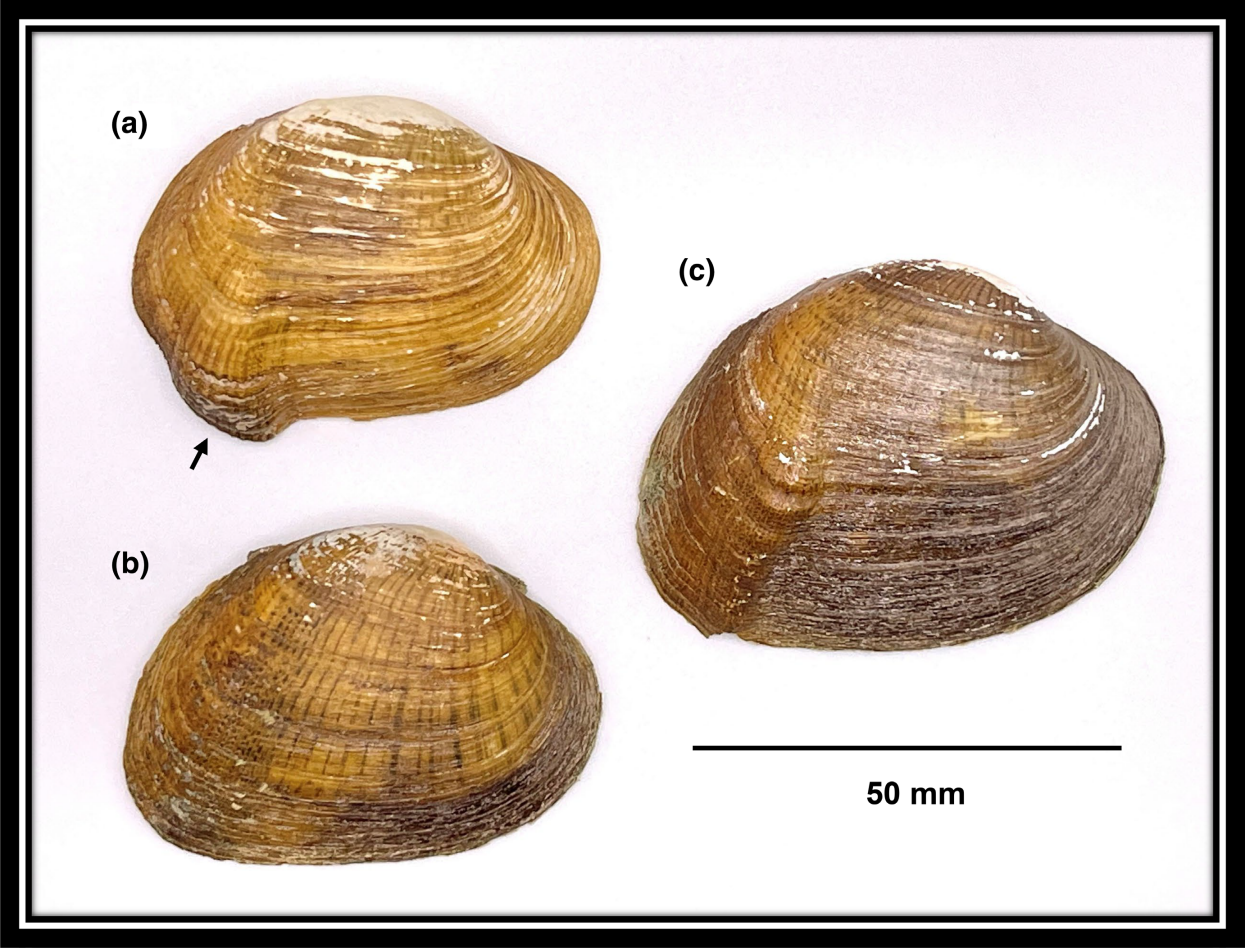
Right valves of Cumberlandian combshell (*Epioblasma brevidens*) showing sexual dimorphism of the female and male shell and oriented with umbo (dorsal) at top, where: (a) is a typical female with a large mantle lure shell expansion (see black arrow near ventral margin) to house both the mantle lure and denticulations on the shell margin to capture host fish; (b) is a typical male with no shell expansion; and (c) is an atypical “male” or “female” shell exhibiting a very diminutive mantle lure shell expansion that seemingly started out as a normal female but then does not develop fully, even recedes as the individual aged, and begins to appear more male‐like. Photographs were taken by Jess Jones.

Some life history traits of *E. brevidens* are known, including the spawning and gravidity periods, and when the females release their larvae (glochidia) to host fishes. Females of *E. brevidens* are long‐term winter brooders, eggs are fertilized in late summer and early fall and the embryos then mature into glochidia in the outer gills of females from early fall through winter for release to host fishes the following spring (Yeager & Saylor, 1995). Like most North American mussel species, *E. brevidens* parasitizes fish with its glochidia to complete its life cycle. The glochidia attach and encyst on the gills, fins, and body of medium to large darters in the genus *Etheostoma* and *Percina*, its natural fish hosts, where they metamorphose to the juvenile stage in 2–3 weeks (perhaps longer) depending on water temperature and then excyst to begin life on the river bottom (Yeager & Saylor, 1995). Average sex ratios in the wild have been reported as approximately 60% male to 40% female and may be influenced by higher predation while females display their mantle lure at the surface of the river bed (Jones & Neves, 2011; Lane et al., 2021). The male and female shells of adults are dimorphic and easily distinguishable from each other, with the ventral margin of the female shell expanded to house the mantle lure (Figure 1). The mantle lure is highly morphologically specialized mantle tissue that mimics prey items of the host fish, and its morphology, behavior, and mimicry in *E. brevidens* is the primary focus of this report.

While not well documented in the scientific literature on the topic of animal mimicry, freshwater mussels arguably exhibit some of the most varied and complex mimicry known in nature. Due to the aquatic habitats that mussels live in, their mimicry has escaped notice by naturalists and biologists until the late 20th and early 21st centuries. However, over the last two decades, biologists have made astonishing discoveries on the mantle lures and conglutinate mimicry utilized by mussels to infest their host fishes with glochidia. Conglutinates are small (typically 5–10 mm long) packets composed of an outer layer or inner core of undeveloped “structural” eggs wherein hundreds to thousands of glochidia are encased or embedded (Barnhart et al., 2008; Jones et al., 2004; Jones & Neves, 2002; Watters, 1999). These conglutinates are released by females and act like miniature “trojan horses,” resembling aquatic insects or other prey items, deceiving fish to feed on them and ultimately to become parasitized with glochidia. The mantle lures and conglutinates of various mussel species can mimic fish, a wide variety of aquatic insects, crayfish, snails, leeches, worms, and other prey of their fish hosts (Barnhart et al., 2008; Haag, 2012; Jones et al., 2004, 2006, 2010; Jones & Neves, 2002).

**FIGURE 2:**
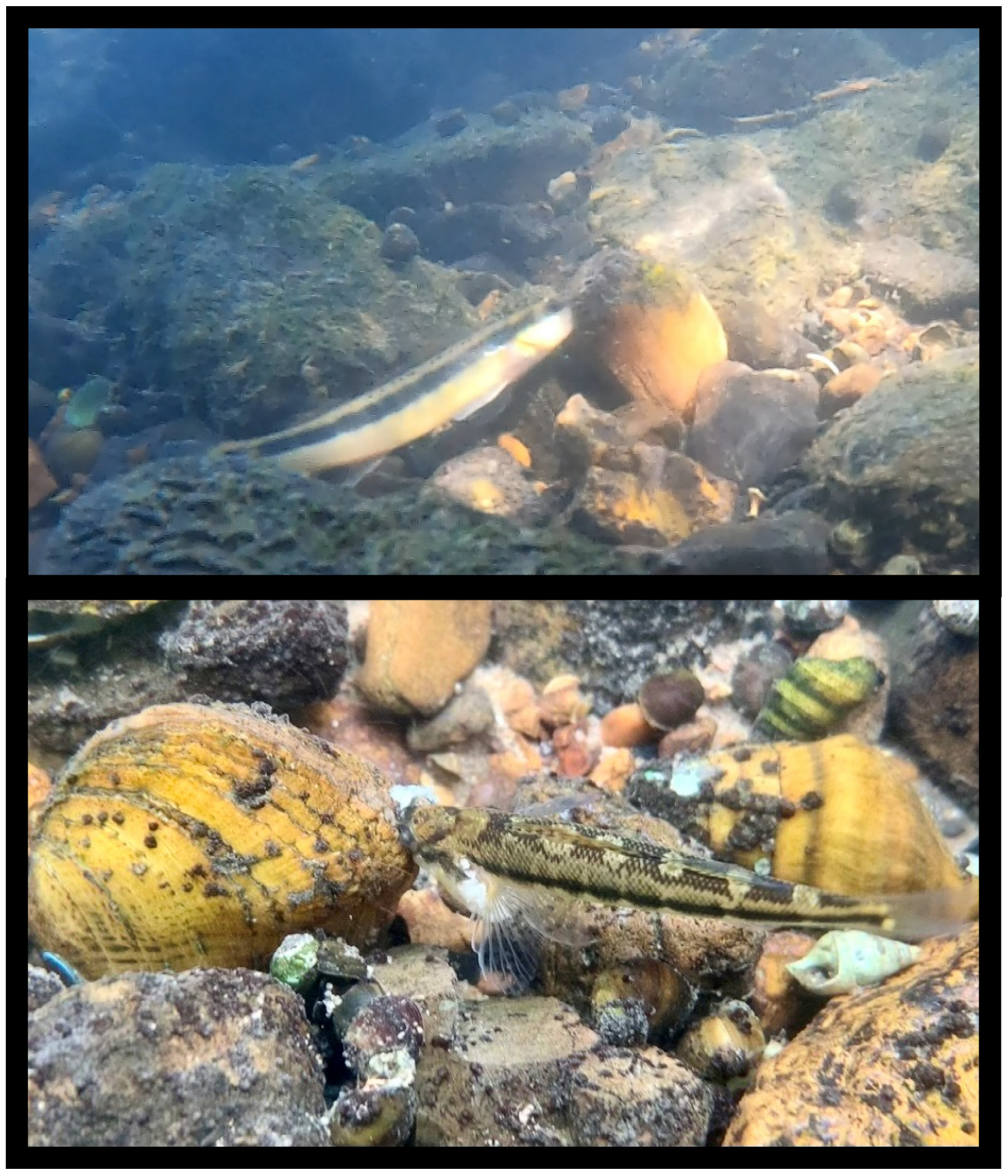
Capture of a Tangerine darter (*Percina aurantiaca*) (Top) and Gilt darter (*Percina evides*) (Bottom) by female *Epioblasma brevidens* in the Powell River, Lee County, Virginia in September 2022. Tangerine darter is ~15 cm long and Gilt dater is ~10 cm long. Top and Bottom photographs taken by Sarah Colletti, Virginia Department of Wildlife Resources.

Perhaps the most behaviorally complex infestation strategy belongs to the *Epioblasma*, where females use their mantle lures to attract and then capture a fish host between their valves like a “venus flytrap.” However, in the case of *E. brevidens*, our observations indicate that only the head rather than the entire body is captured by the female mussel. This allows *Epioblasmas* to infest their glochidia directly on fish (Jones, 2004; Jones et al., 2006). Host capture can last for several minutes and is traumatic to the host fish, which may cause injury and even death of the captured fish (Barnhart et al., 2008). While fish‐capture behavior has been documented in the laboratory under controlled settings for two closely related *Epioblasma* species, it has only recently been seen in *E. brevidens* and these observations were made in the wild. In 2022, a mussel survey was conducted by the Virginia Department of Wildlife Resources (VDWR) and partners in the Powell River at Fletcher Ford, Virginia, where two separate capture events were observed. The first observation was made by Isabel Boyce (Freshwater Mollusk Conservation Center, Virginia Tech, Blacksburg) on September 14 of a captured Tangerine darter (*Percina aurantiaca*), and the second observation was made by Sarah Colletti (VDWR) on September 15 of a captured Gilt darter (*Percina evides*), where both fish were caught by the head and gill operculum by female *E. brevidens* (Figure 2). A timelapse sequence taken with a GoPro9 by John Hartley (Virginia Department of Conservation and Recreation) of the captured tangerine darter can be viewed in Video 1. The total elapsed time from the first observation of the fish being infested to release was 5 min; the initial fish capture was not observed and hence the entire infestation may have lasted longer. A high‐definition video taken by Brittany Bajo‐Walker (VDWR) of the captured gilt dater can be viewed in Video 2. The initial capture of the gilt darter was observed (but not videoed) and the entire infestation lasted 7 min. These are the first observations, photographs, and video recordings of this behavior for the species, and they clearly demonstrate the trauma and perhaps even injury to the fish host.

Video of a female Cumberlandian combshell (*Epioblasma brevidens*) infesting and releasing a host fish, the Tangerine darter (*Percina aurantiaca*). Video was taken by John Hartley (Virginia Department of Conservation and Recreation) at Fletcher Ford on September 14, 2022 in the Powell River, Lee County, Virginia. Video is three seconds long and the time lapse is 5 frames per second and is shown at 1× speed but it can be slowed to 0.25× speed using the Playback speed option menu.

Video of a female Cumberlandian combshell (*Epioblasma brevidens*) capturing and infesting a host fish, the Gilt darter (*Percina evides*). Video was taken by Brittany Bajo‐Walker (Virginia Department of Wildlife Resources) at Fletcher Ford on September 15, 2022 in the Powell River, Lee County, Virginia. Video is 4 min and 34 s long and the time lapse is 30 frames per second and is shown at normal 1× speed.

At its heart, animal and plant mimicry involves deception, where one organism mimics the morphology, behavior, auditory, or chemical cues of another species to dupe (deceive) a third species. Typically, mimicry involves three species, the mimic, the model, and the dupe or what is referred to as the species that receives the signal (i.e., the signal receiver); however, in some mimicry systems, two out of three or all three of the mimic, model, and dupe can be the same species. By deceiving the dupe, the mimic improves its individual fitness, gaining a selective advantage by evading predation, preying on the dupe, or in the case of freshwater mussels, facilitating reproduction. While no universally accepted definition of mimicry exists, Font (2019) recently refined previous definitions with the following: “Mimicry is resemblance/similarity in appearance and/or behavior between a mimic and a model that provides a selective advantage to the mimic because it affects the behavior of a receiver causing it to misidentify the mimic and that evolved (or is maintained by selection) because of those effects.” Hence, in freshwater mussels, the female is the *mimic*, the various prey items that they mimic serve as the *model* organisms, and the *dupe* or *signal receiver* is the host fish. Mussel mimicry can be further categorized as *aggressive mimicry* where the mimic either preys on or parasitizes the dupe and *reproductive mimicry* where the mimic is deceiving the dupe to aid in its reproduction (Table 1). Thus, the purpose of our study was to describe the mantle lure morphology and mimicry of female *E. brevidens*, and report on the novel mantle lure and mimicry we observed in the males.

**TABLE 1:** Types of mimicry possibly exhibited by female and male Cumberlandian combshell (*Epioblasma brevidens*)

Type of mimicry	Male	Female	General description	Specific description
Aggressive		X	Mimic preys on or exploits the signal receiver (dupe) by sharing traits of a harmless species (model).	Female mussel mimics crayfish (model) reproductive traits to lure‐in, capture and parasitize host fish.
Sexual	X		One sex mimicking the opposite sex in behavior, morphological appearance, or chemical signals.	Male mimics female mantle lure.
Reproductive	X	X	Action(s) of the signal receiver (dupe) aid in the mimic's reproduction.	Female mussel mimics crayfish (model) reproductive traits to lure‐in, capture and parasitize host fish to aid in reproduction. Male mussel also mimics crayfish reproductive traits but only to lure‐in host to facilitate reproduction.
Rewarding	X		Mimic signals fitness rewards to manipulate the receiver's behavior	Male mussel mimics crayfish reproductive traits to lure‐in and facilitate reproduction by providing benign or positive signal to fish host.

## 2 METHODS AND RESULTS

**FIGURE 3:**
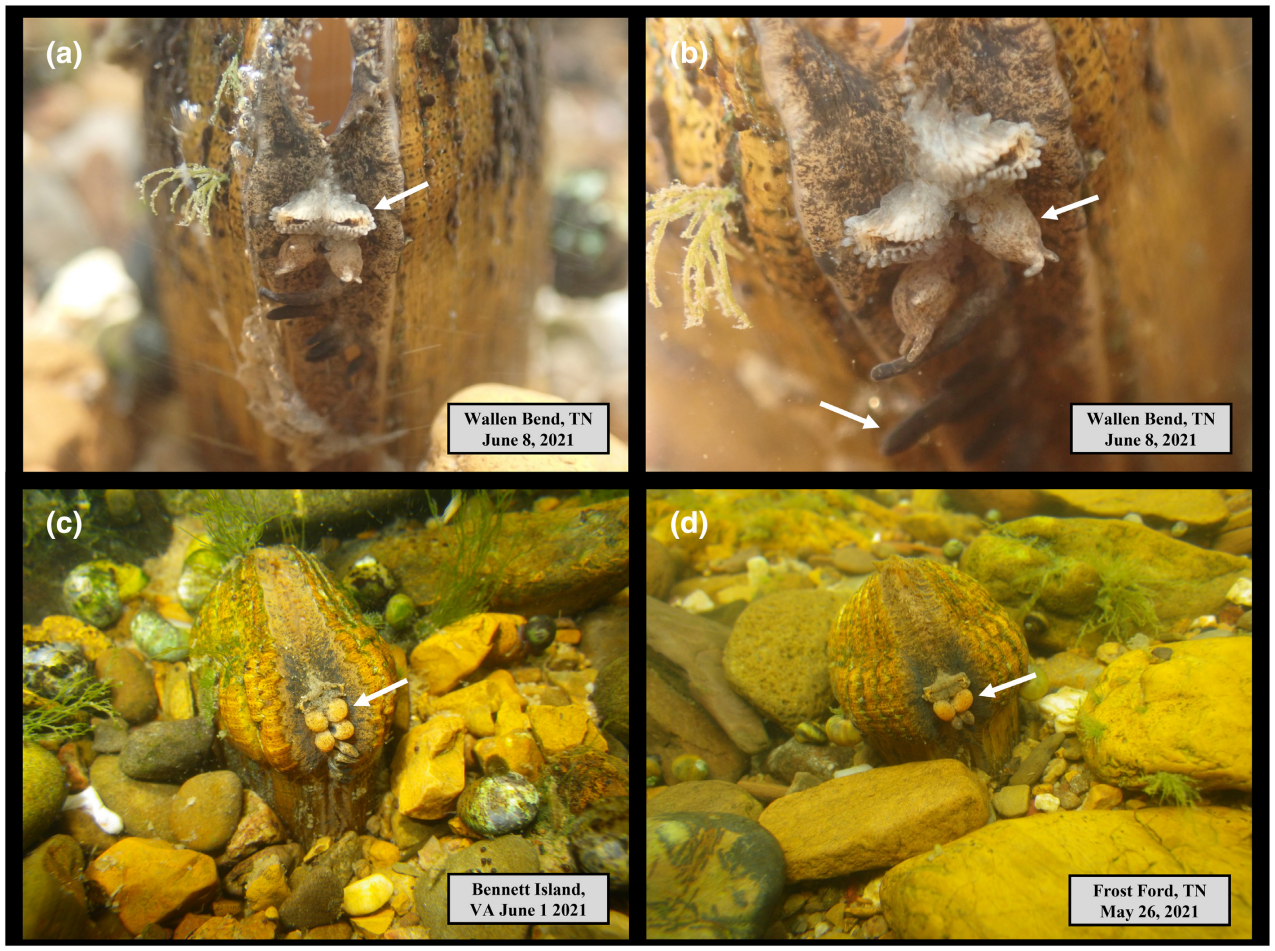
Photographs of the mantle lure displays of female Cumberlandian combshell (*Epioblasma brevidens*) mimicking external reproductive morphology of a female crayfish, where white arrows are pointing to: (a) mimic of crayfish “oviducts”; (b) mimic of crayfish larvae incased in egg membrane (top arrow) and crayfish pleopods (lower arrow); (c) a female mimicking four crayfish eggs; (d) a female mimicking only two eggs and then displaying two larvae immediately below eggs. Photographs a and b are of the same female and notice that this mussel does not have an “egg” display like the females in photographs c and d. All photographs were taken by Jess Jones.

**FIGURE 4:**
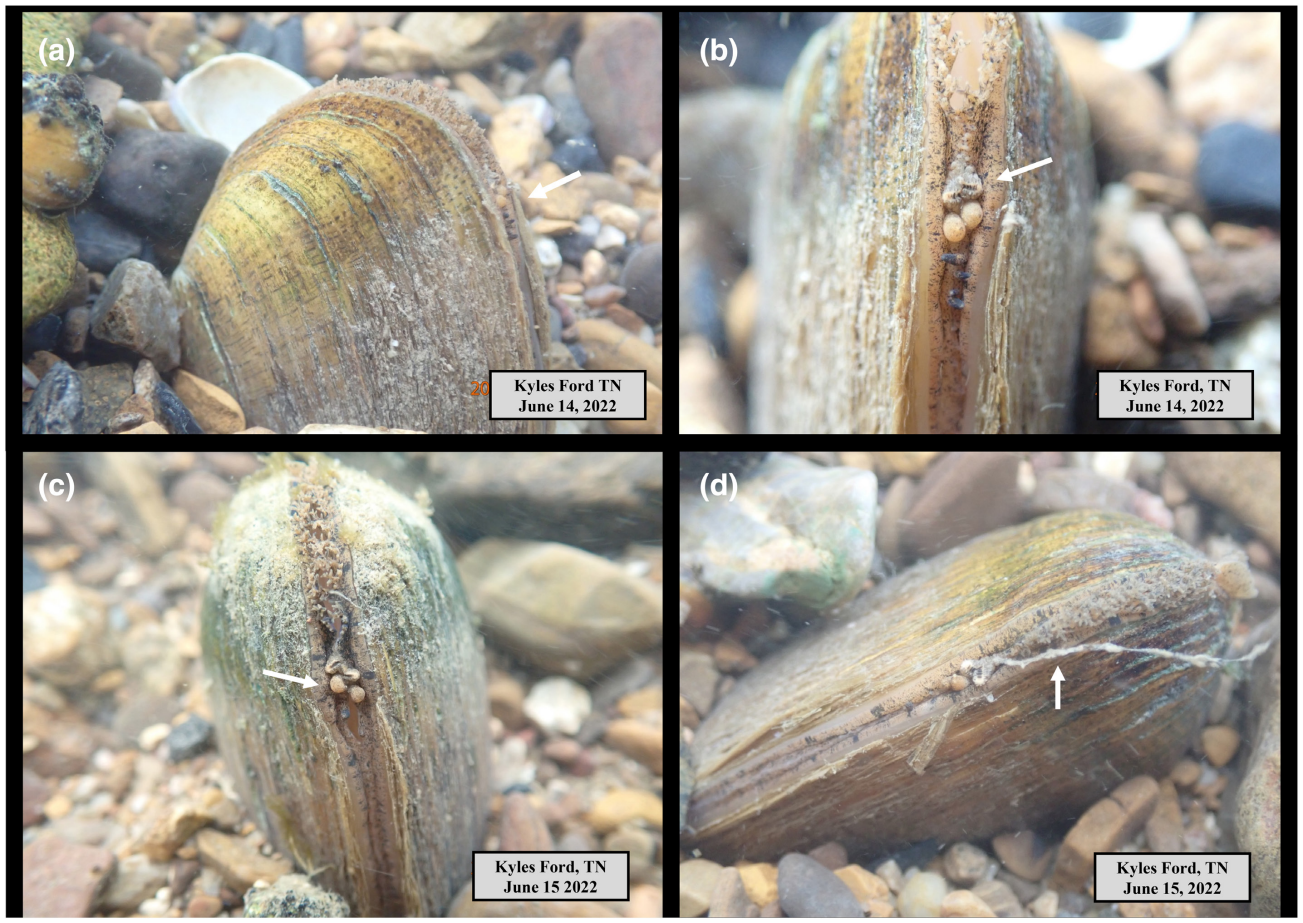
Photographs of the mantle lure displays of male Cumberlandian combshell (*Epioblasma brevidens*) mimicking external reproductive morphology of a female crayfish, where white arrows are pointing to: (a) location on ventral margin of shell where mantle lure is located; (b) mimic of crayfish oviducts; (c) a male mimicking two crayfish eggs; (d) a male mussel releasing a mucus strand from the immediate area of the mantle lure. Photographs a and b are the same individual, but c and d are different individuals. All photographs were taken by Jess Jones.

We conducted mask and snorkel surveys in May and June of 2021 and 2022 to locate, observe, photograph, and video female *E. brevidens* displaying their mantle lures at four sites in the Clinch River: (1) Bennett Island, Russell County, VA (39.9601545, −82.0966773), (2) Wallens Bend, Hancock County, TN (36.5798441, −83.0047563), (3) Kyles Ford, Hancock County, TN (36.5656077, −83.0412957), and (4) Frost Ford, Hancock County, TN (36.5308985, −83.1507785). These sites harbor highly diverse mussel assemblages of >30 species that co‐occur with *E. brevidens* (Jones et al., 2014, 2018). Water temperature during this period ranged between 20 and 24 degrees Celsius. We observed mantle lures from >24 females and 6 males, and all had mantle lures, i.e., of the mussels we observed, we did not see any without a mantle lure. Mantle lures were located on the ventral margin near the incurrent siphon. The mantle lures of female mussels appear to mimic four distinct characteristics of the reproductive anatomy of the underside (ventral) of a female crayfish, to include: (1) the external apertures of the oviducts located on the base of the third pair of walking legs, (2) crayfish larvae still encased in the egg membrane, (3) pleopods or claws, and (4) eggs or embryos (see Figure 3a–d). Females exhibited variation in the number of egg mimics that they displayed, we observed some individuals with four eggs (Figure 3c), another with only two eggs (Figure 3d), one with three eggs (not shown), and one with no eggs (Figure 3a, b). Similarly, mantle lures of male mussels mimicked three characteristics of the female crayfish reproductive anatomy, to include the oviducts, eggs, and/or pleopods and claws (see Figure 4a–c). While we did not directly measure the lengths and diameters of these morphological characters, they generally appeared approximately 2–3 mm smaller in length or diameter than the same characters in females. We did not observe the males mimicking crayfish larvae, but perhaps, this type of mimic does exist in some individuals; we just did not observe them given our limited sample size. We also noticed some of the males releasing short (3–5 mm) to long (>20 mm) mucus strands from near the mantle lure (Figure 4d). Further, the six males we observed clearly were male based on their shell morphology, i.e., no mantle lure shell expansion was present on either valve (Figure 4a). Finally, the mantle lure from the female in Figure 3a, b shows the subtle movement of the oviducts, larvae, and pleopods or claws, indicating that these morphological structures are under the nervous system and muscular control (see Video 3). Photographs in Figures 3 and 4; Video 3 were taken with an Olympus Tough TG‐6 4 K underwater camera by Jess Jones.

Video of a female Cumberlandian combshell (*Epioblasma brevidens*) displaying and moving mantle lure. Video is of the same female mussel shown in Figure 3a, b taken at Wallen Bend on June 8, 2021 in the Clinch River, Hancock County, Tennessee. Video is 28 s long and the time lapse is 30 frames per second and is shown at normal 1× speed.

## 3 DISCUSSION

The mantle lure morphology of female *E. brevidens* is anatomically complex and appears to us to mimic various reproductive traits of a gravid female crayfish. More specifically, it is mimicking food items of its host fish to lure it into close contact so it can capture and infest it with glochidia. It appears to be a form of *aggressive* and *reproductive mimicry* (Table 1). While this mimicry may not appear “perfect,” the eggs and pleopods closely resemble those of an actual crayfish. For example, the pleopods exhibit opening and closing motions, providing greater realism and deception to potential fish hosts, and the eggs look very similar to those carried underneath the abdomen of a gravid crayfish. The brown and mottled color of the eggs is quite similar in appearance and size to the eggs developed by crayfish in the genus *Cambarus*, which commonly co‐occur in streams throughout the distribution of *E. brevidens* (see photographs in Graham & Loughman, 2022). Further, imperfect mimicry in the mind of humans does not mean the mimic is imperfect in the mind of the receiver (McClean et al., 2019). The prey preferences and habits of logperch and other *Percina* darters are not well known but likely are composed of various aquatic insects and other aquatic macroinvertebrates (Jenkins & Burkhead, 1994). Crayfish are abundant in stream ecosystems and are highly nutritious and thus a preferred prey item for many fish species, such as black basses (*Micropterus* spp.), rockbass (*Ambloplites rupestris*), catfish (*Ictalurus* spp.), freshwater drum (*Aplodinotus grunniens*), sculpins (*Cottus* spp.), and many other fish species. Like these and other fish species, consumption of crayfish, especially their eggs and larvae, would be nutritious for logperch as well. Hence, we believe based on our field observations, photographs, and video of displaying females and males, *E. brevidens* in the Clinch River likely is mimicking crayfish as the primary model species, rather than fish eggs as proposed by Barnhart et al. (2008).

The mantle lure morphology of male *E. brevidens* also is anatomically complex and closely resembles the female mantle lure, but comparatively the individual features appear smaller. Male mimicry of the reproductive features of the female is a form of *sexual mimicry*, where one sex mimics the others’ anatomy, and chemical or auditory cues (Table 1). The male lure has oviducts, eggs and pleopods, but they all appear diminutive to those same structures in females. But why do the males have these lures, what are they being used for? Males do not harbor larvae and they do not have the sharp denticulations of the mantle lure shell expansion needed for capturing the fish host. Hence clearly, they are not using it to capture and infect fish with glochidia. We believe one plausible explanation is the male lure is being used to “train” or “desensitize” host fish to increase encounter and capture rates by females. As defined by Jamie (2017), it might serve as a form of *rewarding mimicry* where the mimic signals fitness rewards to manipulate the dupe's behavior. The male is signaling to the fish host that interactions with these lures are harmless, and if the fish feed on the mucus strand, then it could be rewarding the fish when it interacts with the lure. Thus, interactions by the fish host with the male lure could be either benign or positive and may help prime the host to interact more frequently with the female. We hypothesize that mantle lure displaying by males may come with fitness‐related consequences, such as increased energetic demands associated with positioning and displaying at the substrate surface, and injury and predation caused by a range of fish, amphibian, invertebrate, and mammal predators.

By contrast, interactions with female *E. brevidens* would obviously be traumatic if the fish host is captured, hence providing a very negative experience, one that would likely lead to future avoidance. Males then may help offset this negative signal by providing a benign or positive signal. As seen in the gilt darter capture (Video 2), the mucous strands produced by the female mussel while capturing and infesting the darter are similar to those produced by males (Figure 4d) and could be used as a reward to lure‐in nearby schooling fish. Thus, the gilt darter captured by the female mussel may not be the sole target because the fish may ultimately not survive the encounter. A copious amount of mucous containing glochidia can be seen surrounding the captured fish, which may attract other nearby darters to consume it and become infected. Gilt darters and other confirmed hosts of *E. brevidens* are curious by nature and often school and aggressively compete over small prey items (Jett, 2010).

We describe for the first time the mantle lure mimicry of *E. brevidens*, showing its close resemblance to the reproductive anatomy of a gravid female crayfish, and a novel form of mimicry in males. To our knowledge, mantle lures in males have not been previously documented in freshwater mussels. While this appears to be a form of sexual mimicry, it is also atypical in that the deception of the mimic is not being used to gain an immediate mating advantage over the individual. But rather through rewarding mimicry males are providing a reproductive advantage at a broader level for the population of female *E. brevidens*. Other mussel species also use logperch as host, such as snuffbox (*Epioblasma triquetra*) (Yeager & Saylor, 1995). Thus, this rewarding mimicry strategy may aid in the direct competition for fish hosts with congeners.

Finally, we close by acknowledging areas of uncertainty to hopefully encourage future research. First, mussels are long‐lived and how the mantle lure may change morphologically over time as the animal ages are unknown. For example, do the mantle lures of females add eggs or other features as they age, perhaps going from not having eggs when they are young to having them as they age? Or perhaps eggs or other features could be removed, torn off by fish hosts interacting with the mantle lure? Second, hermaphroditism is well known in various unionid taxa in North America (Downing et al., 1989; Heard, 1975, 1979; Kat, 1983; Van der Schalie, 1970), and has even been documented in *Epioblasma* spp. in the Clinch River (Henley, 2019). Females for example can produce both eggs and sperm in their gonadal tissue and hence self‐fertilize, commonly known as simultaneous hermaphroditism. Perhaps the males with mantle lures that we observed are beginning to change into females, and thus represents a type of sequential hermaphroditism that includes both gonadal and morphological sexual changes. Shell characteristics exemplified in extreme examples of *E. brevidens* may demonstrate this phenomenon, where hormonal changes are driving changes in the shell phenotype. For example, we have observed individuals of this species that appear initially to be female based on the external shell morphology but exhibit only a small mantle lure shell expansion that never fully develops, and as the individual ages, the shell becomes more male‐like (Figure 1c). And vice versa, individuals with shells that were clearly male but developed the mantle lure shell expansion much later in adulthood. Hence, hermaphroditic features as expressed in the shell and mantle lure phenotype may be more prevalent in this species than previously understood.

## AUTHOR CONTRIBUTIONS

**Zachary Taylor:** Investigation (supporting); methodology (supporting); resources (supporting); validation (supporting); visualization (supporting); writing – review and editing (equal). **Timothy Lane:** Investigation (supporting); methodology (supporting); resources (supporting); writing – review and editing (equal). **Jess W. Jones:** Conceptualization (lead); investigation (lead); methodology (lead); resources (lead); supervision (lead); validation (equal); visualization (equal); writing – original draft (lead); writing – review and editing (equal).

## CONFLICT OF INTEREST STATEMENT

The authors declare no conflict of interest.

## Data Availability

All data collected during this field study are presented in the photographs (Figures 1, 2, 3, 4) and videos ([Link], [Link], [Link]) provided in this published article. No additional data will be uploaded to another data repository.
